# Use of Soft Cervical Collar among Whiplash Patients in Two Italian Emergency Departments Is Associated with Persistence of Symptoms: A Propensity Score Matching Analysis

**DOI:** 10.3390/healthcare9101363

**Published:** 2021-10-14

**Authors:** Firas Mourad, Giacomo Rossettini, Erasmo Galeno, Alberto Patuzzo, Giuseppe Zolla, Filippo Maselli, Federica Ciolan, Michele Guerra, Giacomo Tosato, Alvisa Palese, Marco Testa, Giorgio Ricci, Arian Zaboli, Antonio Bonora, Gianni Turcato

**Affiliations:** 1 Department of Physiotherapy, LUNEX International University of Health, Exercise and Sports, 4671 Differdange, Luxembourg; 2 Luxemburg Health & Sport Sciences Research Institute A.s.b.l., 50, Avenue du Parc des Sports, 4671 Differdange, Luxembourg; 3 Department of Clinical Science and Translation Medicine, University of Rome Tor Vergata, 00133 Roma, Italy; eragal@me.com (E.G.); albertoalberto01@gmail.com (A.P.); zollagiuseppe@gmail.com (G.Z.); 4 School of Physiotherapy, University of Verona, 37129 Verona, Italy; giacomo.rossettini@gmail.com; 5 Polimedico Specialistico STEMA Fisiolab, 04100 Latina, Italy; 6 Centro Riabilitativo Agorà Medical, 37057 Verona, Italy; 7 Studio Fisioterapico BiàMed, 03043 Cassino, Italy; 8 Department of Neuroscience, Rehabilitation, Ophthalmology, Genetics, Maternal and Child Health, Campus of Savona, University of Genoa, 17100 Savona, Italy; masellifilippo76@gmail.com (F.M.); marco.testa@unige.it (M.T.); 9 Sovrintendenza Sanitaria Regionale Puglia INAIL, 70126 Bari, Italy; 10 Rehabilitation Unit, Azienda ULSS n.9 Scaligera, San Bonifacio, 37047 Verona, Italy; federicaciolan@gmail.com; 11 Rehabilitation Unit, Azienda ULSS n. 9 Scaligera, Villafranca Di Verona, 37069 Verona, Italy; michele-guerra@virgilio.it; 12 Rehabilitation Unit, Clinica San Francesco, 37127 Verona, Italy; giacomo.tosato@hotmail.it; 13 Department of Medical Sciences, University of Udine, 33100 Udine, Italy; alvisa.palese@uniud.it; 14 Emergency Department, University Hospital of Verona, 37126 Verona, Italy; giorgio.ricci@aovr.veneto.it (G.R.); antonio.bonora@aovr.veneto.it (A.B.); 15 Emergency Department, General Hospital of Merano (SABES-ASDAA), 39012 Merano, Italy; zaboliarian@gmail.com (A.Z.); gianni.turcato@yahoo.it (G.T.)

**Keywords:** cervical collar, whiplash injuries, physical therapy, emergency department, neck

## Abstract

Purpose: Although the use of soft cervical collars in the emergency department (ED), for whiplash-associated disorders (WAD), is controversial, it is still widely adopted. The purpose of our study was to investigate the impact of the early use of soft cervical collars on the return to the ED, within three months of a road traffic collision. Methods: We conducted a retrospective observational study on WAD patients from two EDs in Verona (Italy). Patients in the earlier acute phase of WAD (within 48 h from the trauma) were included; those with serious conditions (WAD IV) were excluded. As an end point, we considered patients who returned to the ED complaining of WAD symptoms within three months as positive outcome for WAD persistence. Results: 2162 patients were included; of those, 85.4% (n = 1847/2162) received a soft cervical collar prescription. Further, 8.4% (*n* = 156/1847) of those with a soft cervical collar prescription, and 2.5% (*n* = 8/315) of those without a soft cervical collar (*p* < 0.001) returned to the ED within three months. The use of the soft cervical collar was an independent risk factor for ED return within three months, with an OR, adjusted for possible clinical confounders, equal to 3.418 (95% CI 1.653–7.069; *p* < 0.001). After the propensity score matching, 25.5% of the patients (*n* = 25/98) using the soft cervical collar returned to the ED at three months, compared to the 6.1% (*n* = 6/98) that did not adopt the soft cervical collar. The use of a soft cervical collar was associated with ED return with an OR = 4.314 (95% CI 2.066–11.668; *p* = 0.001). Conclusions: Our study shows that the positioning of the soft collar in a cohort of patients with acute WAD, following a rear-end car collision, is an independent potential risk factor to the return to the ED. Clinically, the use of the collar is a non-recommended practice and seems to be related to an increased risk of delayed recovery. There is a need to inform healthcare providers involved in the ED of the aim to limit the use of the soft cervical collar. A closer collaboration between clinicians (e.g., physicians, physical therapists, nurses) is suggested in the ED. Future primary studies should determine differences between having used or not having used the collar, and compare early physical therapy in the ED compared with the utilization of the collar.

## 1. Introduction

The Quebec Task Force defined whiplash-associated disorders (WAD) as an umbrella term, which includes “a variety of clinical manifestations following an acceleration–deceleration injury commonly resulting from a road traffic collision”, recommending a four-grade classification system based on severity (Table 1) [1]. The worldwide economic burden of WAD is huge and differs across countries, also depending on healthcare-systems and insurance refunding [2,3]. Emergency department (ED) accesses for WAD have been reported to have an annual incidence ranging from 70 per 100,000 inhabitants in Quebec to 325 per 100,000 in the Netherlands, with an average of 235–300/100,000 [4,5,6]. In Italy, the most recent report has estimated the number of WADs to be 12,235 cases per year, with a higher incidence in the northern regions [7].

The early presentation of acute WAD varies widely and may include symptoms such as the following: cervical pain; headache; shoulder or arm pain; limitation of neck movements; jaw pain; dizziness; nausea; tinnitus; memory loss; concentration difficulties [8,9,10]. Generally, half of WADs recover within 2–3 months; while the remaining 50% continue to present symptoms, resulting in a delayed recovery [11,12]. Accordingly, whiplash injuries may be a manifestation of real, but not yet widely recognized or acknowledged, physical injuries to the soft tissues of the head and neck [13]. Also, the interaction between the neck and the midbrain has been recently suggested as a cause of the persistence of symptoms. That is, recent studies have documented the relevance of a central pathophysiological adaptation mechanism, as WAD is frequently associated with post-traumatic syndrome, panic attacks, anxiety, kinesiophobia, pain spreading, disability, and a reduced quality of life [11,14,15]. These latest psychological components have been shown to be strong predictors for a delayed recovery [16,17,18,19].

Early and appropriate management of WAD is of paramount importance for both the prevention of delayed recovery and the development of risk factors. However, there is a lack of evidence on the best practice that healthcare providers should adopt in the ED, as first-line treatment [9]. Most of the studies available include acute WAD patients (within 15 days from the trauma) with heterogeneous interventions (e.g., pharmacological, education and reassurance, exercise, manual therapy, spinal manipulation, immobilization, acupuncture, and rest) and follow-up (referral to general practitioner, returned to work, physiotherapy, etc.) [20]. Within this context, the use of soft cervical collars remains an empirical widespread practice in ED [21]. However, the evidence on its use is conflicting [20,22,23,24], leading the Quebec Task Force to recommend the avoidance of its use for WAD I-II because it provides no adjunctive benefit [1].

Although methodological concerns that may have a direct impact on the interpretation of the findings arose [25], Ricciardi et al., in their systematic review, concluded that a non-immobilization protocol for WAD has better outcomes in relieving pain and in the recovery of neck function [26]. In a more recent systematic review, Christenesen et al. confirmed that an active/act-as-usual approach has more favorable outcomes in terms of pain reduction [27]. These findings increase the attention on the soft cervical collar as a contributor to the persistence of symptoms. Although the short-term use of the soft collar, in the very early stages, has been suggested to not have detrimental effects on the overall outcome [28], to date, only a few studies consider the soft cervical collar as an earlier acute intervention (time 0—within 48 h from the whiplash trauma), and present methodological limitations (e.g., the small cohort), providing poor results on the effectiveness of the soft cervical collar in a very early phase of WAD [20,29]. Therefore, the management of the WAD in the first 48 h continues to represent a conundrum for healthcare providers.

Thus, our study aims to investigate whether the use of soft cervical collars in an earlier acute phase of WAD 0–III increases the risk of symptoms persisting, leading to a return to the ED within three months of a road traffic collision. To the best of the authors knowledge, this is the first study including WAD 0–III patients in very early stage, within the 48 h from their whiplash injury.

## 2. Methods

### 2.1. Study Design

We conducted a retrospective multicenter observational study following the Strengthening the Reporting of Observational studies in Epidemiology guidelines [30] at the EDs of two hospitals in Verona, Italy, namely, the Ospedale Civile Maggiore (90,000 visits per year) and the Policlinico Universitario di Verona, Italy (50,000 visits per year). We performed this study in compliance with the principles outlined in the Declaration of Helsinki. Ethics approval was obtained from the Ethics Committee for Clinical Trials, Verona, Italy (approval number: 889CESC-2018).

### 2.2. Patients Eligibility Criteria

All ED records of patients who accessed an ED evaluation for road traffic collision, presenting symptoms of WAD between January 2013 and December 2014, were initially extracted from the computer database using the dedicated FirstSTATA software.

Through manual evaluation of the extracted records, a team of three physiotherapist experts in the management of WAD and two physicians with more than 5 years of experience in emergency medicine were involved. We only included patients with a WAD 0–III due to an acute (within 48 h of ED presentation) posterior or lateral car accident [1].

All patients who did not meet the inclusion criteria were excluded if they presented any of the following [24,31,32,33,34,35,36]: WAD IV (e.g., due to fracture); a head injury associated with WAD (e.g., concussion); a delayed access to the ED (e.g., 48 h after injury); a mechanism of injury other than motor vehicle accident (e.g., motorbike or bicycle accident, pedestrian accident, accidental fall, or axial load on the head); an age younger than 14 years and older than 85 years. Moreover, all patients with incomplete records or unavailable follow-up were excluded.

### 2.3. Dataset Creation

The dataset was created recording the most common features of WAD presented in literature [1,34,35,36,37,38], such as the following: the condition of trauma (e.g., dangerous mechanism, airbag blast, and acute intoxication); the most common symptoms (e.g., alteration of consciousness, neck pain, related thoracic and/or low back pain, limbs paresthesia); the clinical findings (e.g., midline cervical spine tenderness, cervical paravertebral muscle tenderness, upper trapezius tenderness, pain during active cervical rotation, and active cervical rotation <45° degrees); the grade of WAD [1,34,35,36,37,38]. Moreover, we collected the following features from ED medical records: age; gender; the time of access in ED (e.g., within 3 h, within 12 h, and over 12–48 h).

### 2.4. Intervention and Outcomes

The prescription and placement of a soft cervical collar at discharge from both EDs was recorded for each patient from the study database. Accordingly, the following two groups of patients were retrospectively identified: (a) patients with soft cervical collar; (b) patients without soft cervical collar.

The patients’ management with WAD in both EDs was homogeneous and reported as follows: (a) patients accessed the ED; (b) patients performed the medical triage; (c) patients were assessed by the emergency physician; (d) the patients underwent diagnostic imaging (i.e., radiography); (e) patients were re-assessed by the emergency physician who decided whether or not to prescribe the soft cervical collar based on his discretional experience as the emergency physician responsible for each patient.

The primary outcome of the study was the return to ED within three months. The need for a further visit due to WAD symptoms (e.g., neck pain, headache, shoulder or arm pain, dizziness, tinnitus, memory and concentration difficulties or psychological disorders, such as post-traumatic syndrome, sleep disturbance, panic attack) [8,9,10,39,40,41,42] was considered a positive outcome. Despite the fact that patients may have had different options of outpatient care for their symptoms (e.g., general physician visit, private specialist consultation), we considered the return to ED within 3 months as a surrogate of severity, as an emergency visit is the option with the greatest care intensity. The outcome was identified by assessing all medical records available in the computer database for each patient enrolled in the study by two expert emergency physicians.

### 2.5. Data Processing and Analysis

The categorical variables were expressed as a percentage; while the continuous variables were reported as a median and interquartile range (IQR). The univariate comparisons between the clinical variables, recorded at the time of the access to the ED, and the outcomes were conducted with a Fisher exact test, a chi-square test or the Mann–Whitney test when appropriate.

The possible independent association between the soft cervical collar application and the outcome of the study (returning to ED for post-traumatic WAD symptoms) was investigated by performing a multivariate model adjusted for clinical and anamnestic variables previously found to be significant in the univariate analyses and included as possible multivariate confounders. The multivariate model was run through a binary logistic regression and the independent association between WAD application and return to the ED was described in terms of adjusted odd ratio (OR). The 95% confidence intervals were reported.

In addition, considering the possible bias due to a non-homogenous therapeutic choice of applying the soft cervical collar by an emergency physician, the clinical variables of the patients collected during the visit to the ED, which may have influenced the decision for prescribing the soft cervical collar, were used to create propensity score matching. Propensity score matching is a retrospective sampling statistical technique that can limit the selection bias of two study groups affected by a possible initial incorrect sampling [43,44] such as, in the current case, the physician’s arbitrary decision to apply the soft cervical collar.

Propensity score matching allowed a subgroup of patients divided 1:1 between the two treatment groups (e.g., collar versus non-collar) to be obtained, balanced by their anamnestic and clinical variables (differences between the variables were set with a *p* > 0.1).

After propensity score matching, the univariate and multivariate analyses were repeated to estimate the independent association between the use of the soft cervical collar and the risk of returning to the ED. All analyses were considered significant for a *p* < 0.05 and were conducted with the statistical software STATA 13.0 (StataCorp, College Station TX, USA).

## 3. Results

### 3.1. Baseline Characteristics and Clinical Variables

**Figure 1:**
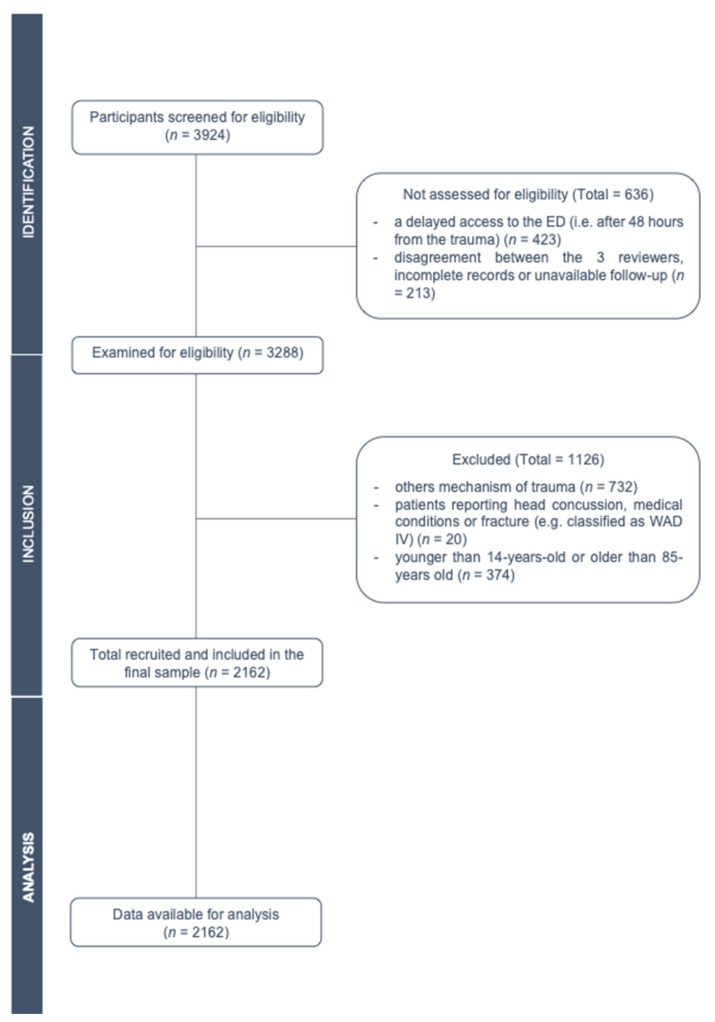
Flow diagram of eligible subjects according to STROBE statement.

During the 24-month study period, 2162 eligible WAD patients were identified (1164 females, median age = 38 years, IQR 28–50). A flow chart, with the participant selection process, is summarized in Figure 1. Of those patients, 4.5% (*n* = 98/2162) were classified as WAD 0; 47% (*n* = 1017/2162) as WAD I; 45.7% (*n* = 989) as WAD II; 2.7% (*n* = 58) as WAD III.

The soft cervical collar was applied in 85.4% (*n* = 1847/2162) of WAD patients, while 14.6% (*n* = 315/2162) did not receive the soft cervical collar.

The baseline characteristics and clinical variables recorded at admission are reported in Table 2. The most relevant clinical variables associated with soft cervical collar prescription were as follows: gender (*p* < 0.001); alteration of consciousness (*n* = 10, 0.5%; *p* = 0.002); acute intoxication (*n* = 10, 0.5%; *p* < 0.001); neck pain (*n* = 1765, 95.6%; *p* < 0.001); tenderness of the trapezius muscles (*n* = 687, 37.2%; *p* < 0.001); pain during active cervical rotation (*n* = 473; 25.6%; *p* = 0.048); dangerous collision mechanism (*n* = 136; 7.4%; *p*-value = 0.018).

In addition, an increase in severity (expressed as degree of WAD) was associated with a greater likelihood of receiving a soft cervical collar (*p* < 0.001).

### 3.2. Variable Associated with a Return within 3 Months to the ED

An overall rate of 7.6% (*n* = 164/2162) of patients with WAD returned to the ED within three months. Among the group of patients with a soft cervical collar prescription, 8.4% (156/1847) returned to the ED within three months, compared with 2.5% (8/315) of those without a soft cervical collar (*p* < 0.001).

The clinical characteristics recorded upon patient arrival to the ED, according to their return to the ED within three months, or not, are listed in Table 3.

Gender (*p*-value = 0.004); tenderness of the trapezius muscle (*n* = 71, 43.4%; *p*-value = 0.016); pain during active cervical rotation (*n* = 59, 36%; *p*-value = 0.001); active cervical rotation <45 degrees (*n* = 28, 17.1%; *p*-value < 0.001); dangerous collision mechanism (*n* = 24, 14.6%; *p*-value = 0.002); thoracic/lumbar pain (*n* = 78, 47.6%; *p*-value = 0.006); the grade severity of WAD (*p*-value = 0.003); the use of the soft cervical collar (*n* = 156, 95.1%; *p*-value > 0.001); and the days of soft cervical collar positioning (median = 8, IQR = 6–10; *p*-value = 0.022) were all significantly associated with a return to the ED within 3 months.

The multivariate analysis, comparing soft cervical collar application and the risk of returning to the ED within three months, adjusted for all possible clinical variables that were statistically significant in the previous univariate analysis, confirmed a strong independent association between the use of the soft cervical collar and the risk of WAD symptoms persisting (adjusted OR = 3.418, 95% CI 1.653–7.069, *p* < 0.001).

### 3.3. Propensity Score Matching for Soft Cervical Collar Application and for the Return to the ED

The clinical variables were unbalanced between the two study groups. Soft cervical collar vs. no cervical collar, with a *p* < 0.1 (Table 4), were used to calculate the propensity score matching for every patient. With a caliper of 0.01, statistical matching, using the propensity score of the patients, was performed, resulting in 98 pairs of patients, perfectly divided between treated with a soft cervical collar (*n* = 98) and without a cervical collar (*n* = 98). In this new cohort of 196 patients, none of the clinical characteristics recorded at the time of ED admission were found to be distributed differently in the two treatment groups (Table 4). Further, 15.8% (31/196) of the patients presented another ED admission within three months. Of these, 80.6% of the patients (*n* = 25/31) were treated with a soft cervical collar, while the remaining 19.4% (6/31) were treated without a cervical collar (Table 5).

The following multivariate analysis, adjusted for WAD grade, showed that the application of a soft cervical collar was an independent risk factor for subsequent admission to the ED in the propensity group as well (adjusted OR = 4.314, 95% CI 2.066–11.668, *p* = 0.001). Multivariate analysis also confirmed, in the cohort of patients obtained by limiting possible initial selection bias, that soft cervical collar placement is an important risk factor for subsequent admissions, regardless of the status and clinical severity of the patient in the ED, expressed by the severity surrogate “WAD grade”.

## 4. Discussion

Evidence-based clinical practice guidelines did not recommend the use of a soft cervical collar [4,9,45] because of the lack of clinical evidence supporting its benefit [10,46,47]. However, in practice, the soft cervical collar is still widely used (85.4%) for WAD patients in two Italian EDs. Although the quality of the evidence is limited due to the limitations and the retrospective design of our study, we observed that the use of a soft cervical collar in an earlier acute WAD (within 48 h from the trauma) was significantly associated with a return to the ED within three months, compared to the group of patients with no soft cervical collar positioning, independently of the WAD severity. Thus, our findings are aligned with the guidelines recommendations [4,9,45], suggesting that the non-immobilization approach and early active management may reduce the risk of returning to the ED and the possible persistence of WAD.

In this study, we used the return to the ED as an outcome. This methodological choice was used to control the several treatment options that a patient after WAD could have in an outpatient setting (e.g., general practitioner visit, private specialist consultation). Our choice to favor an ED visit may indicate a higher level of acuity. Thus, we considered patients requesting a second ED visit as those presenting a more severe clinical condition.

As emerged from our study, clinicians decided to prescribe the cervical collar mainly in accordance with the most relevant physical impairments presented by patients when accessing the ED, such as the following: neck pain, tenderness of the trapezius muscles, and pain during active cervical rotation. While tissue damage could explain the symptoms in the early stage, the cervical collar may influence psychosocial factors, which may contribute to a delayed recovery [48,49,50]. Psychological and behavioral factors following a traffic collision, such as post-traumatic stress, pain catastrophizing, low self-efficacy, passive coping, kinesiophobia, and avoiding activities due to a fear of pain, are consistently reported as being associated with poor outcomes in WAD patients [11,12,16,18,19].

Considering the physical impairments presented in WAD patients, recent studies found that during a collision—especially at low speed—the neck muscles (e.g., the sternocleidomastoid, trapezius, and erector spinae muscles) strongly react prior to the peak head acceleration, influencing the dynamic response of the head–neck complex and the whiplash mechanism injury [51,52]. The muscle’s capacity to perform strength and endurance has been shown to still be impaired after one year from the whiplash injury, leading to severe and persistent disability, even if the neck mobility and pain proprioception recover [48]. Although the mechanism is still unclear, these patients show a significant association with fat infiltration and atrophy in the cervical muscles [53,54]. This secondary maladaptive process is well described in the literature, especially after immobilization, and it is attributed to the combination of both the use of a cervical collar and psychological behavioral factors [53]. Preliminary evidence also suggests that the central mechanism—primarily driven by cognitive behavioral aspects and immobilization—may play a role in the reduction in the alpha motor neuron drive, leading to secondary muscular changes and altered function [55].

The parameters of the whiplash mechanism itself (e.g., the impact direction, the seating positing, the head resting position, and the vehicle status during the collision) have little impact on the prognosis and choices of care (including the use of a soft cervical collar) [56,57]. On the other hand, patients presenting with fear-avoidance beliefs and kinesiophobia are intimately related to persistent disability [18,58,59]. Early intensive care and collar utilization in those patients with a benign profile (e.g., WAD I–III) may reinforce these negative beliefs, facilitating behavioral changes and muscular maladaptation [59,60,61]. The current evidence recommends early management of these negative prognostic factors as first-line treatment, moving the clinicians to emphasize active, rather than passive, treatments. As reported in the literature, multimodal care, including structured education, advice to stay active, exercise (e.g., range of motion and strengthening graded exercise, both supervised and home-based), and time-limited manual therapy, is the most cost-effective strategy for WAD [3,24,46,62,63]. Educating the patients on the benefits of remaining active, moving their neck early, and performing regular therapeutic exercises has also been shown to have a hypoalgesic effect [64]. In addition, cognitive and behavioral treatment strategies seem to be the most promising management strategy for minimizing the risk of the persistence of symptoms in patients with WAD [56].

The notion that active mobilization is superior to collar immobilization has been known since 1994 [31], that is, the majority of the literature on this topic concluded that active strategies (e.g., early exercises, acting similarly to before the trauma, “behave as usual”) are associated with a quicker recovery or, at worst, reveal no differences with early immobilization [22,29,31,32,65,66,67,68,69,70]. In addition, it has also been shown that immobilizing the neck with collars, in healthy individuals, contributes to significantly reducing the neck’s active range of motion in all directions [60].

Although the overall quality of the evidence was low, and the majority of the relevant randomized controlled trials on the topic [45,61,71,72] lack blinding [73] and are affected from affection bias [74], the two most recent systematic reviews did not observe any advantages of an immobilization protocol by the use of a soft collar [27]. Notably, non-immobilization of the neck showed a greater pain intensity reduction [27] and function recovery at the long-term follow-up [26]. Interestingly, these results seem to be independent from the adjunct of physical therapy, highlighting the negative influences of the collar on the non-physical components and, then, the patients’ prognosis [26]. These results agree with our finding, which was as follows: the collar was associated with increased healthcare utilization [15,75,76], leading to possible addictive costs for the national healthcare systems. Therefore, it is a priority to identify appropriate and cost-effective management strategies for WAD [77].

The inconsistent outcomes in the published literature, and the complexity behind the clinical presentation of WAD patients, underline a gap in how healthcare providers effectively manage these patients and limit the long-term negative consequences [63,78]. Moreover, WAD is a compensable injury in many countries, leading to concerns on incentives, behaviors, and outcomes that may arise when financial compensation and legal professionals responsibilities/risks are available [79]. Within this context, patients suffering of WAD sought treatment from a variety of healthcare providers (e.g., physical therapists, chiropractors, physicians) and different settings (e.g., public and private) [80]. As a result, clinical practice and management strategies might be deeply influenced, leading to a conflicting practice to the current guidelines [80]. The utilization of the soft cervical collar seems to not only be related to the perceptions of effectiveness by both the patients and the providers, but the prescription and use of the cervical collar and radiography have been documented as being strongly influenced by payment policies; they determine higher refunds, reinforcing the cultural expectation of a worse clinical outcome [81]. Therefore, de-implementing collar use seems to be associated with motivational, economic, political, and legal factors/considerations in care delivery [82,83]. Providing updated evidence-based interventions requires a cultural shift in how clinicians may better influence WAD patients’ outcomes [80]. Accordingly, there is a need to inform the health providers, in order to implement the current guidelines routinely, with the final goal to promote early access to active treatment approaches for WAD patients (e.g., exercises), as well as to objectivize the associated impairments [81] and address the negative prognostic factors (e.g., fear avoidance, maladaptive beliefs) [82,84]. However, for certain well-selected patients with WAD [85,86,87,88], and, accordingly, for patient clinical presentations and preferences, an intermittent use of the collar for a short period and during the acute stage could be potentially indicated when appropriately combined with education and active approaches [24,53,70]. To encourage Italian public administrators and national council representatives, we created and implemented, in the article, an infographic based on the latest guidelines for public use (Supplement 1 in Supplementary Materials), where we suggest a road map based on our previous experience in an Italian ED, where musculoskeletal specialized physical therapists were commissioned to train the staff and organize a management change in patients with WAD [84].

### Strengths and Limitations

As a strength, we strictly excluded all the patients with head trauma, concussion, and WAD IV, with the final attempt to only include WAD 0–III [89]. Although propensity score matching significantly reduced the included subject’s number, it provides a more homogeneous final sample [43,44]. Furthermore, the statistical methodology—propensity score matching and adjustment of the results based on the WAD severity at the time of the ED visit—strengthens our results.

However, our study presented several limitations. Firstly, a retrospective study could suffer from structure bias [90]; secondly, no information on adjunctive treatments associated with the cervical collar (e.g., pharmacology management, physical therapy) was collected. Another limitation is the possible application of a not-soft cervical collar, which was a discretional physician’s choice. Moreover, the follow-up at three months prevents the patients’ clinical information being obtained for the long term (e.g., six months). Furthermore, the selected outcome, restricted to “emergency departments return”, does not allow all those patients who seek treatment outside of the ED (e.g., private settings, clinics) to be identified. Finally, the patient sample collected was dated (years 2013–2014) and, although WAD management in Italy is widely based on a single clinician’s expertise, guidelines and local laws have been changed [84,91].

## 5. Conclusions

Our study retrospectively shows that the positioning of the soft cervical collar in a cohort of patients with acute WAD, following a rear-end car collision, is an independent potential risk factor for returns to the ED. Clinically, the use of the collar is a non-recommended practice and seems to be related to an increased risk of delayed recovery. The management of WAD in the acute phase should include earlier multimodal care with structured education, advice to stay active, and exercise. Future primary studies should determine differences between having used or not having used the collar (e.g., observational prospective), and compare early physical therapy in the ED with collar utilization (e.g., randomized controlled trial).

## Figures and Tables

**Table 1:** Quebec Task Force whiplash-associated disorders (WAD) classification.

Grade	Clinical Presentation
**0**	No complaint about neck pain No physical signs
**I**	Neck complaint of pain: Stiffness or tenderness only Non-physical signs
**II**	Neck complaint Musculoskeletal signs, including: Decreased range of motion Point tenderness
**III**	Neck complaint Neurological signs, including: Decreased or absent tendon reflexes Muscles weakness Sensory deficits
**IV**	Neck complaint and fracture or dislocation

**Table 2:** Baseline characteristics and clinical variables.

Variables	Soft Cervical Collar	No Soft Cervical Collar	*p*-Value
Patients, *n* (%)	1847 (85.4)	315 (14.6)	
Age, years, median (IQR)	40 (28–52)	38 (27–49)	0.026
Gender, *n* (%) Female Male	133 (42.2) 182 (57.8)	1031 (55.8) 816 (44.2)	<0.001 *
Time of access in ED, *n* (%) Within 3 h Within 12 h Over 12–48 h	1001 (54.2) 223 (12.1) 620 (33.6)	178 (56.5) 42 (13.5) 95 (30.1)	0.200
Dangerous mechanism of trauma, *n* (%)	36 (11.4)	136 (7.4)	0.018 *
Trauma related to airbag blast, *n* (%)	630 (34.1)	106 (33.6)	0.901
Trauma related to acute intoxication, *n* (%)	12 (3.8)	10 (0.5)	<0.001 *
Alteration of the consciousness at the first evaluation in ED, *n* (%)	8 (2.5)	10 (0.5)	0.002 *
Neck pain at the first evaluation in ED, *n* (%)	267 (84.8)	1765 (95.6)	<0.001 *
Thoracic/Lumbar pain at the first evaluation in ED, *n* (%)	122 (38.7)	686 (37.1)	0.614
Limbs paresthesia at the first evaluation in ED, *n* (%)	6 (1.9)	51 (2.8)	0.453
Presence of midline cervical spine tenderness at CE, *n* (%)	118 (37.5)	648 (35.1)	0.444
Presence of cervical muscles tenderness at CE, *n* (%)	93 (29.5)	596 (32.3)	0.360
Presence of upper trapezius tenderness at CE, *n* (%)	56 (17.8)	687 (37.2)	<0.001 *
Presence of pain during active cervical rotation at CE, *n* (%)	64 (20.3)	473 (25.6)	0.048 *
Presence of active cervical rotation <45 degrees at CE, *n* (%)	14 (4.4)	135 (7.3)	0.070
Grade of WAD ^1^, *n* (%) 0 I II III	45 (14.3) 152 (48.3) 112 (35.6) 6 (1.9)	53 (2.9) 865 (46.8) 877 (47.5) 52 (2.8)	<0.001 *

^1^ Refer to Table 1 for further details. Abbreviations: ED, emergency department; IQR, interquartile range; *n*, number; CE, clinical evaluation; WAD, whiplash-associated disorders; %, percentage; *, statistically significant.

**Table 3:** Variable associated with a return within three months to the emergency department.

Variables	No Return	Return within 3 Months	*p*-Value
Patients, *n* (%)	1998 (92.4)	164 (7.6)	
Age, years, median (IQR)	38 (27–50)	38 (29–48)	0.821
Gender, *n* (%) Female Male	1058 (53) 940 (47)	106 (64.6) 58 (35.4)	0.004 *
Use of soft cervical collar, *n* (%)	1691 (84.6)	156 (95.1)	<0.001 *
Days of soft cervical collar positioning, median (IQR)	7 (5–8)	8 (6–10)	0.022 *
Dangerous mechanism of trauma, *n* (%)	148 (7.4)	24 (14.6)	0.002 *
Trauma related to airbag blast, *n* (%)	661 (33.1)	64 (39)	0.220
Trauma related to acute intoxication, *n* (%)	20 (1.0)	2 (1.2)	0.681
Alteration of the consciousness at the first evaluation in ED, *n* (%)	16 (0.8)	2 (1.2)	0.642
Neck pain at the first evaluation in ED, *n* (%)	1876 (93.9)	156 (95.1)	0.675
Thoracic/Lumbar pain at the first evaluation in ED, *n* (%)	730 (36.5)	78 (47.6)	0.006 *
Limbs paresthesia at the first evaluation in ED, *n* (%)	51 (2.6)	6 (3.7)	0.440
Presence of midline cervical spine tenderness at CE, *n* (%)	716 (35.8)	50 (30.5)	0.175
Presence of cervical muscles tenderness at CE, *n* (%)	640 (32.0)	49 (29.9)	0.602
Presence of upper trapezius tenderness at CE, *n* (%)	672 (33.6)	71 (43.3)	0.016 *
Presence of pain during active cervical rotation at CE, *n* (%)	478 (23.9)	59 (36.0)	0.001 *
Presence of active cervical rotation <45 degrees at CE, *n* (%)	121 (6.1)	28 (17.1)	<0.001 *
Grade of WAD ^1^, *n* (%) 0 I II III	94 (4.7) 952 (47.6) 901 (45.1) 51 (2.6)	4 (2.4) 65 (39.6) 88 (53.7) 7 (4.3)	0.003 *

^1^ Refer to Table 1 for further details. Abbreviations: IQR, interquartile range; *n*, number; CE, clinical evaluation; WAD, whiplash-associated disorders; %, percentage; *, statistically significant.

**Table 4:** Propensity score matching for collar application.

Variables	No Soft Cervical Collar	Soft Cervical Collar	*p*-Value
Patients, *n* (%)	98 (50.0)	98 (50.0)	
Age, years, median (IQR)	45 (28–54)	36 (23–51)	0.055
Gender, *n* (%) Female Male	47 (48.0) 51 (52.0)	57 (58.2) 41 (41.8)	0.126
Dangerous mechanism of trauma, *n* (%)	11 (11.2)	18 (18.4)	0.227
Trauma related to airbag blast, *n* (%)	51 (51.9)	44 (45.0)	0.625
Trauma related to acute intoxication, *n* (%)	9 (9.2)	7 (7.1)	0.795
Alteration of the consciousness at the first evaluation in ED, *n* (%)	8 (8.2)	7 (7.1)	1.000
Neck pain at the first evaluation in ED, *n* (%)	87 (88.8)	84 (85.7)	0.669
Thoracic/Lumbar pain at the first evaluation in ED, *n* (%)	47 (48.0)	42 (42.9)	0.566
Limbs paresthesia at the first evaluation in ED, *n* (%)	6 (6.1)	5 (5.1)	1.000
Presence of midline cervical spine tenderness at CE, *n* (%)	43 (43.9)	46 (46.9)	0.776
Presence of cervical muscles tenderness at CE, *n* (%)	37 (37.8)	44 (44.9)	0.384
Presence of upper trapezius tenderness at CE, *n* (%)	26 (26.5)	29 (29.6)	0.751
Presence of pain during active cervical rotation at CE, *n* (%)	43 (43.9)	47 (48.0)	0.667
Presence of active cervical rotation <45 degrees at CE, *n* (%)	13 (13.3)	13 (13.3)	1.000
Grade of WAD ^1^, *n* (%) 0 I II III	9 (9.2) 24 (24.5) 59 (60.2) 6 (6.1)	6 (6.1) 32 (32.7) 54 (55.1) 6 (6.1)	0.585

^1^ Refer to Table 1 for further details. Abbreviations: IQR, interquartile range; *n*, number; CE, clinical evaluation; WAD, whiplash-associated disorders; %, percentage.

**Table 5:** Propensity score matching for the return to the emergency department.

Variables	No Return	Return within 3 Months	*p*-Value
Patients, *n* (%)	165 (84.2)	31 (15.8)	
Age, years, median (IQR)	40 (25–54)	42 (28–46)	0.373
Gender, *n* (%) Female Male	86 (52.1) 79 (47.9)	18 (58.1) 13 (41.9)	0.563
Positioning of soft cervical collar, *n* (%)	73 (44.2)	25 (80.6)	<0.001 *
Days of soft cervical collar positioning, median (IQR)	0 (0–7)	7 (3–9)	<0.001 *
Dangerous mechanism of trauma, *n* (%)	20 (12.1)	9 (29.0)	0.025
Trauma related to airbag blast, *n* (%)	69 (42.1)	25 (80.0)	0.039
Trauma related to acute intoxication, *n* (%)	14 (8.5)	2 (6.5)	0.753
Alteration of the consciousness at the first evaluation in ED, *n* (%)	13 (7.9)	2 (6.5)	1.000
Neck pain at the first evaluation in ED, *n* (%)	145 (87.9)	26 (83.9)	0.559
Thoracic/Lumbar pain at the first evaluation in ED, *n* (%)	74 (44.8)	15 (48.4)	0.844
Limbs paresthesia at the first evaluation in ED, *n* (%)	10 (6.1)	1 (3.2)	1.000
Presence of midline cervical spine tenderness at CE, *n* (%)	78 (47.3)	11 (35.5)	0.245
Presence of cervical muscles tenderness at CE, *n* (%)	71 (43.0)	10 (32.3)	0.322
Presence of upper trapezius tenderness at CE, *n* (%)	42 (25.5)	13 (41.9)	0.080
Presence of pain during active cervical rotation at CE, *n* (%)	72 (43.6)	18 (58.1)	0.170
Presence of active cervical rotation <45 degrees at CE, *n* (%)	19 (11.5)	7 (22.6)	0.143
Grade of WAD ^1^, *n* (%) 0 I II III	14 (8.5) 47 (28.5) 94 (57.0) 10 (6.1)	1 (3.2) 9 (29.0) 19 (61.3) 2 (6.5)	0.856

^1^ Refer to Table 1 for further details. Abbreviations: IQR, interquartile range; *n*, number; CE, clinical evaluation; WAD, whiplash-associated disorders; %, percentage; *, statistically significant.

## Data Availability

Raw data are available upon request.

## Supplementary Materials

The following are available online at https://www.mdpi.com/article/10.3390/healthcare9101363/s1, Supplement 1: the impact of early collar utilization in whiplash patients in emergency department: move forward.
