# Supplementary material for: Use of Soft Cervical Collar among Whiplash Patients in Two Italian Emergency Departments Is Associated with Persistence of Symptoms: A Propensity Score Matching Analysis
Source: Healthcare (Basel). 2021 Oct 14;9(10):1363. doi: 10.3390/healthcare9101363 (PMC8544415; doi:10.3390/healthcare9101363)
Supplement: Supplementary file 1 [file healthcare-09-01363-s001.zip › healthcare-1399625-supplementary.pdf]

THE IMPACT OF AN EARLY COLLAR UTILIZATION IN WHIPLASH PATIENTS IN EMERGENCY DEPARTMENT: MOVE FORWARD

1

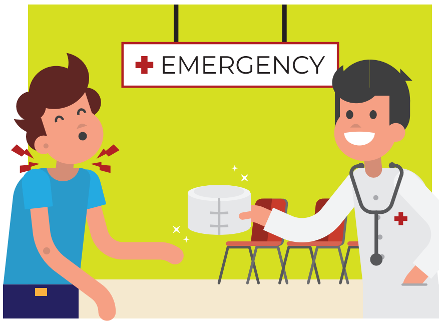

- Although the use of the collar in whiplash patients is controversial, it is still widely used and left to emergency physician discretion;

- We found that the cervical collar use is significantlly (95% CI 2.066 - 11.668, p=0.001) associated with emergency department return (OR=4.314).

2

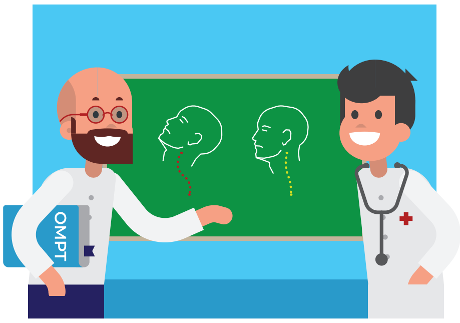

- The collar utilization is not recommended as it may reinforce negative beliefs facilitating behavioral changes and maladaptation;[1-3]

- An inter-disciplinary continuous professional development course based on recommendation of the most recent literature has the potential to improve the efficiency of Emergency Department without affecting the patients' safety.[4]

3

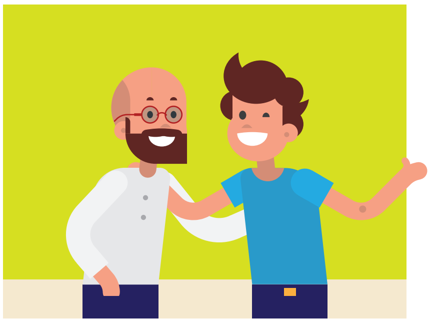

- Guidelines recommend as first-line treatment: address negative prognostic factor, reassure and advice to stay active, exercises, time-limited musculoskeletal physical therapy.[5-9]

BIBLIOGRAPHY

1. Courtney DM. Assessment and management of whiplash from the emergency and acute care setting: Care, questions, and future global research needs. J Orthop Sports Phys Ther. 2016;46(10):822-825. doi:10.2519/jospt.2016.0114

2. Barati K, Arazpour M, Vameghi R, et al. The Effect of Soft and Rigid Cervical Collars on Head and Neck Immobilization in Healthy Subjects. Asian Spine J. 2017;11(3):390-395. doi:10.4184/asj.2017.11.3.390

3. Gross AR, Kaplan F, Huang S, et al. Psychological Care, Patient Education, Orthotics, Ergonomics and Prevention Strategies for Neck Pain: An Systematic Overview Update as Part of the ICON§ Project. Open Orthop J. 2013;7(1):530-561. doi:10.2174/1874325001307010530

4. Mourad F, Patuzzo A, Tenci A, et al. Management of whiplash-associated disorder in the Italian emergency department: the feasibility of an evidence-based continuous professional development course provided by physiotherapists. Disabil Rehabil. 2020:1-8. doi:10.1080/09638288.2020.1806936. Published online August 27

5. Van der Velde G, Yu H, Paulden M, et al. Which interventions are cost-effective for the management of whiplash-associated and neck pain-associated disorders? A systematic review of the health economic literature by the Ontario Protocol for Traffic Injury Management (OPTIMa) Collaboration. Spine J. 2016;16(12):1582-1597. doi:10.1016/j.spinee.2015.08.025

6. Fritz J. Toward improving outcomes in Whiplash: Implementing new directions of care. J Orthop Sports Phys Ther. 2017;47(7):447-448. doi:10.2519/jospt.2017.0107

7. Côté P, Wong JJ, Sutton D, et al. Management of neck pain and associated disorders: A clinical practice guideline from the Ontario Protocol for Traffic Injury Management (OPTIMa) Collaboration. Eur Spine J. 2016;25(7):2000-2022. doi:10.1007/s00586-016-4467-7

8. Wong JJ, Côté P, Shearer HM, et al. Clinical practice guidelines for the management of conditions related to traffic collisions: a systematic review by the OPTIMa Collaboration. Disabil Rehabil. 2015;37(6):471-489. doi:10.3109/09638288.2014.932448

9. Bussières AE, Stewart G, Al-Zoubi F, et al. The Treatment of Neck Pain-Associated Disorders and Whiplash-Associated Disorders: A Clinical Practice Guideline. J Manipulative Physiol Ther. 2016;39(8):523-564.e27. doi:10.1016/j.jmpt.2016.08.007
